# Alternatives to robenidine to control gastrointestinal disorders of weaner rabbits in the field

**DOI:** 10.1016/j.vas.2021.100179

**Published:** 2021-05-27

**Authors:** Clarissa A. Jung, Paul P. Torgerson, Roger Bolt, Felix Grimm, Julia Schädler, Sarah Albini, Annette Liesegang

**Affiliations:** a Institute of Animal Nutrition, Vetsuisse Faculty, University of Zurich, Winterthurerstrasse 270, 8057 Zurich, Switzerland; b Section of Epidemiology, Vetsuisse Faculty, University of Zurich, Winterthurerstrasse 270, 8057 Zurich, Switzerland; c Department for livestock husbandry, Strickhof, Eschikon 21, 8315 Lindau, Strickhof, Switzerland; d Institute of Parasitology, Vetsuisse Faculty, University of Zurich, Winterthurerstrasse 266a, Switzerland; e National Reference Centre for Poultry and Rabbit Diseases, Institute for Food Safety and Hygiene, Vetsuisse Faculty, University of Zurich, Winterthurerstrasse 270, 8057 Zurich, Switzerland

**Keywords:** *Eimeria* sp., *E. coli*, Feed additives, Coccidiostats, Gastrointestinal disorders, Weaner rabbits

## Abstract

Gastrointestinal disorders due to *Eimeria* sp. and *E. coli* overgrowth cause high mortality in weaner rabbits and the interest in alternatives to coccidiostats is high. This study aimed to investigate the superiority of natural feed additives towards robenidine preserving gastrointestinal health in the field. Rabbits were divided into four groups, Control Group (CG) exclusively supplemented with robenidine, Sainfoin Group (SG) was supplemented with a combination of robenidine and sainfoin, and two additional groups were respectively supplemented with Herb-All COCC-X (garlic; conessi tree) (HG: Herbal Group) and by a combination of Herb-All COCC-X and Klinofeed (clinoptilolite) (MG: Mineral Group). *Eimeria* sp*.* (98,40%) and *E. coli* overgrowth (73.60%) could be confirmed as the main causes for losses. High mortality rates (SG: 30.00% - MG: 47.50%), also in the groups receiving robenidine (SG: 30.00%; CG: 45.00%), reinforced the importance of alternatives in the field. The natural additives of groups SG, HG and MG did not have a significant influence on the weight gains and the oocyst counts in the jejunum/ileum and caecum of slaughter rabbits at the end of the trial, compared to group CG. Significantly higher oocyst shedding in SG (p = 1.4E-03) and HG (p = 1.4E-05) during the trial may be explained by a higher surviving rate of diseased rabbits in those groups, fostered by beneficial effects of the additives, which should be investigated further.

## Highlights

High mortality rates and oocyst excretion are found in weaner rabbits.
Gastrointestinal disorders have multifactorial origin.
Coccidiostats are commonly used in most rabbitries.
Improve understanding of the use of alternatives to robenidine in the form of natural supplements.

## 1 Introduction

The main reasons for juvenile mortality in rabbits after weaning are digestive disorders accompanied by abdominal distension and diarrhoea. *Eimeria* sp. and an overgrow of bacterial agents, such as *E. coli*, are often observed in those cases (Licois, 2004; Rashwan & Marai, 2000). Due to coccidiosis the intestinal pH is increased, which allows the proliferation of *E. coli,* drawing some attention on the parasite for the control of digestive disturbances in rabbit weaners (Barthold, Griffey & Percy, 2016).

Weaning is a critical period for the sensitive young rabbits and the basis for the prevention of diseases is hygienic and proper housing (Rashwan & Marai, 2000). Anticoccidials, which are commonly used prophylactically to prevent losses due to digestive disorders in rabbit meat production, face rising resistances and low acceptance in public (Pankandl et al., 2009). Thus, alternative dietary measures for preventing intestinal diseases in fattening rabbits become attractive.

Sainfoin (*Onobrychis viciifolia)* is an interesting alternative due to its high content of tannins, resulting in astringent, abirritative, anti-inflammatory, slightly local anaesthetic and antimicrobial effects (Liu et al., 2013; Teuscher, Melzig, Matthias & Lindequist, 2012). Beneficial effects on the growth rates, *E. coli* and *Eimeria*-infection rates of fattening rabbits are reported (Dalle Zotte & Cossu, 2009; Legendre et al., 2018).

There have also been several studies reporting on the attenuating effect of garlic (*Allium sativum*) on intestinal coccidiosis in rabbits (Kowalska, Bielański, Nosal & Kowal, 2012; Nosal, Kowalska, Bielański, Kowal & Kornás, 2014). Furthermore, garlic possesses antimicrobial (also against *Escherichia* species), antioxidative and anti-inflammatory effects (Al-Quraishy et al., 2011; Ankri & Mirelman, 1999; Iciek, Kwiecień & Włodek, 2009). Allicin has been pointed out as the most important active compound. However, other authors argue, that the effects of garlic are mediated by synergistic activities amongst different agents (Bayan, Koulivand & Gorji, 2014; Iciek et al., 2009).

Traditionally, conessi tree (*Holarrhena antidysenterica)* is used in ayurvedic medicine for several indications, due to the large number of active principles (Sinha et al., 2013). Documented antibacterial properties (*in vitro*), against *E. coli*, mainly ascribed to the alkaloid conessine, are of special interest for the current study (Siddiqui et al., 2012). In addition to that, relaxing, anti-inflammatory, antioxidant and protective effects on the intestinal mucosa were also described (Darji Vinay, Deshpande Shrikalp & Bariya Aditi, 2012; Gilani et al., 2010; Johari & Gandhi, 2016; Sujan Ganapathy, Ramachandra & Padmalatha Rai, 2011; Xie et al., 2018).

Apart from plants, there has also been research on the health promoting effects of the mineral clinoptilolite. Besides its capability of binding toxins, also an inhibitory effect on *E. coli* was observed (Prasai et al., 2017; Schneider, Zimmermann & Gewehr, 2017; Wu, Wang, Zhou, Zhang & Wang, 2013), which makes it a promising further addition for fighting intestinal diseases in rabbits.

In the BTS guidelines (“Besonders Tierfreundliche Stallhaltungssysteme” – “particularly animal-friendly housing systems”;(Anonymous, 2008; Anonymous, 2013) of the Swiss government, group housing, allowing close contact between the individual animals, is mandatory for fattening rabbits. Thus, the knowledge about the prevalence of distinct pathogens and effective control measures is especially important. It is hypothesised, that digestive disorders, induced by *Eimeria* sp. and *E. coli*, are the main cause for mortality in the first four weeks after weaning in fattening rabbits housed according to the BTS guidelines in a big commercial rabbitry in Switzerland. The study aimed to show an improvement of gastrointestinal health and mortality rates by the inclusion of the described natural supplements in the diets, compared to an exclusive management with robenidine in weaner rabbits.

## 2 Materials and methods

### 2.1 Animals and setting of the trial

**Fig. 1:**
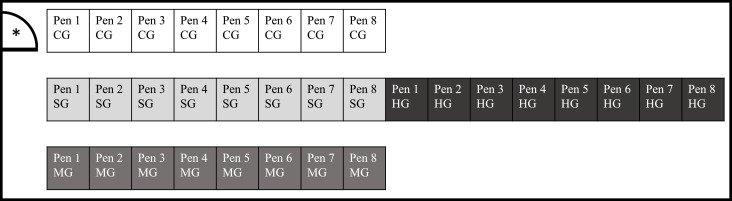
Position of the feeding groups within the stable. The rabbits of the four feeding groups (80 rabbits/group) were housed in subgroups of 10 rabbits/pen (8 pens/group). The pens of CG and MG were located alongside the walls of the stable, while SG and HG were housed in the middle of the stable. CG = Control Group; SG = Sainfoin Group; HG = Herbal Group; MG = Mineral Group; * = door to the vestibule of the stable.

The study was conducted as formally approved by the Veterinary office of the Canton of Aargau (License no. 75724). Housing conditions in the rabbitry conformed to the so called BTS guidelines of the Swiss government (Anonymous, 2013). During the study, rabbits were housed in groups, according to the Animal Welfare Ordinance, article 64 (Anonymous, 2008). For the study, 320 Hycole x Hyla rabbits (aged 25 days, directly after weaning) were used, originating from two different breeding farms. The animals were randomly assigned into four different groups (80 rabbits/group), with a random distribution of sexes, each group composed of rabbits from both suppliers. The animals were housed in subgroups of ten rabbits in pens of 2.17 m^2^ (Control Group (CG); Mineral Group (MG)) to 2.34 m^2^ (Sainfoin Group (SG); Herbal Group (HG)). Each pen was composed of different levels. In the upper levels (0.91 m^2^ in CG and MG; 1.14 m^2^ in SG and HG) straw was used as bedding, to provide higher comfort for the animals. The ground floor was made of plastic slats, reducing the amount of litter in the pens (Fig. 1).

The trial started simultaneously to the weaning of the rabbits (at the age of 25 days) and lasted for 31 days (until the age of 55 days).

### 2.2 Feeding strategy

All groups received a diet composed of fresh water (nipple drinkers), hay, silage and a commercial pelleted fattening feed (UFA AG, Herzogenbuchensee, Switzerland). Amongst groups, only the additives in the pellets and the types of silage differed. In CG and SG the pellets contained the coccidiostat robenidine (65 mg/kg feed). While in HG the pellets were enriched with the herbal mixture “Herb-All COCC-X” (0.3%), a powder mainly consisting of dried garlic and conessi tree (LifeCircle Nutrition, Wangen, Switzerland). The pellets of MG contained clinoptilolite (0.5%) (“Klinofeed”, Unipoint AG, Ossingen, Switzerland), in addition to the herbal mixture (0.3%). Contrary to the other groups, SG received sainfoin silage. The rabbits received the diets *ad libitum* (Table 1).

**Table 1:** Composition of the diets (*ad libitum*) of the four different feeding groups.

	Fresh water	HHay	Types of silage	Supplements of the fattening feed
CG	✓	✓	Alfalfa^1^ silage	65 mg Robenidine/kg feed
SG	✓	✓	Sainfoin^2^ silage	65 mg Robenidine/kg feed
HG	✓	✓	Alfalfa silage	0.3% herbal mixture^3^
MG	✓	✓	Alfalfa silage	0.3% herbal mixture 0.5% clinoptilolite

Table 1 shows the composition of the diets of the four different feeding groups. All components of the diets were fed *ad libitum*.

1 *Medicago sativa*.

2 *Onobrychis viciifolia*.

3 The main ingredients of the herbal mixture are garlic (*Allium sativum*) and conessi tree (*Holarrhena antidysenterica*); CG, Control Group; SG, Sainfoin Group; HG, Herbal Group; MG, Mineral Group.

The described feeding was introduced at the beginning of the trial simultaneously to weaning, at the age of 25 days, and was not changed until the end of the study, at the age of 55 days. Before weaning, the rabbits also had access to fresh water, hay, alfalfa silage and pelleted feed (UFA AG).

### 2.3 Weighing of rabbits

During the study, the rabbits were examined individually and weighed on day 1, 8, 15, 22 and 29 of the trial. General assessment of health and housing of the groups was done by the staff of the rabbitry on a daily basis.

### 2.4 Laboratory examinations

#### 2.4.1 Necropsy, bacteriology and parasitology

Rabbits dying during the trial were collected and stored at +2 °C until dissection, which took place once a week to assess the cause of death. Microbiological samples of the contents of jejunum, ileum and caecum were collected of every dissected rabbit under sterile conditions. The samples were cultured anaerobically on Columbia agar with 7% sheep blood and aerobically on bromothymol blue-lactose agar (Oxoid/Thermo Fisher Scientific, Waltham, MA, USA). Both agars were incubated for 24 h at 37 °C. Bacterial identification was done using the Biotyper MALDI-TOF-MS system (Bruker Daltonics, Billerica, MA, USA/Software: Compass flexControl Version 3.4; MBT Compass 4.1.80). An overgrowth of *E. coli* was defined by the predominant growth of more than 100 colony forming units of *E. coli* per plate, with no or fewer than 10 colonies of concurrent bacterial growth. Furthermore, bacteriological examinations of pathological conspicuous tissues were done using Columbia agar with 7% sheep blood and bromothymol blue-agar, both cultivated aerobically for 24 h at 37 °C (in order to detect other pathogens which might be relevant in the examined weaning period).

Parasitological examination of the gall bladder was done by using a direct smear of its contents on a glass slide. Slides were assessed microscopically with a 100x magnification using a semiquantitative scheme. Four different scores were used to describe the number of oocysts in the smear samples: No oocyst per field of view, one oocyst per field of view, up to ten oocysts per field of view and more than ten oocysts per field of view.

Furthermore, combined parasitological samples of the jejunum/ileum contents and a sample of the caecum content were examined with the *Mc Master* method, as described below. The sample size was adjusted to 1 g, respectively.

On the last day of the study (day 54 of life; day 30 of the study), one animal of each pen was sacrificed in the slaughterhouse of the rabbitry, according to the Swiss regulation (Anonymous, 2010). After stunning with a captive bolt pistol, the animals were sacrificed by exsanguination. These rabbits were also dissected, microbiologically and parasitologically examined, as described above.

#### 2.4.2 McMaster technique for coccidian enumeration (OPG: oocysts per gram faeces)

Pooled faecal samples of each pen were collected for each week of the trial and assessed according to a modified *Mc Master* method with a sensitivity of 50 oocysts per gram faeces, as described below (Perim & Hansen, 1998).

Of each faecal sample, 3 g were mixed with 45 ml of saturated NaCl solution. The suspension was sieved, mixed and transferred to the *Mc Master* chambers. After a flotation time of five minutes the samples were examined microscopically (magnification 100x).

The number of oocysts per gram faeces (OPG) was calculated according to Deplazes, Eckert, von Samson-Himmelstjerna and Zahner (2013):

$$\begin{array}{c} OPG=\frac{counted\;oocysts\;of\;two\;count\;fields}{amount\;of\;faeces\;\left(g\right)\;x\;size\;of\;count\;field\;\left(cm^{2}\right)}x\\ \frac{volume\;of\;the\;suspension\;(ml)}{hight\;of\;the\;chamber\;(cm)\;x\;number\;of\;the\;count\;fields} \end{array}$$

Samples with higher parasitic loads were further diluted and the formula was adapted adequately.

#### 2.4.3 Detection of Salmonella

Faecal samples of 25 g, collected per individual pen on day 15 of the study were cultured according to the ISO method (Anonymous, 2017). Briefly, samples were incubated in buffered peptone water (“Buffered Peptone Water (ISO)”, Oxoid/Thermo Fisher Scientific) for 24 h at 37 °C, subcultivated onto modified semi-solid Rappaport Vassiliadis medium (Merck, Darmstadt, Germany) and incubated at 41.5 °C for 18 h. Suspected swarming halos were subcultivated onto xylose lysine deoxycholate agar and Brilliance™ Salmonella agar (Oxoid/Thermo Fisher Scientific).

### 2.5 Statistical evaluation

All analyses were undertaken in R (R Foundation for Statistical Computing, 2019 (R Core Team, 2019). Differences were considered significant at a value of *p* ≤ 0.05. The OPG counts of the faecal and intestinal samples were highly aggregated with the variance of counts much greater than the mean. There were also repeated measures from the housing as single boxes. Hence analysis required a negative binomial generalized linear mixed model. With OPG the dependant variable, single groups as a fixed factor and the housing in single boxes as a random effect. For this analysis the glner.nb function from the lme4 package in R was used (Bates, Maechler, Bolker & Walker, 2015). The weight gain of the different groups during the trial was normally distributed and hence was analysed with a linear mixed model. Again, the fixed factor was defined as the division in the single groups and the different weeks of the experiment and the boxes used analysed as a random slope and intercept respectively. For this analysis the lmer function, also from the lme4 package in R, was used. For the oocyst counts in the caecum and jejunum at post mortem a generalized linear model was used with no random effects as there was no repeat measures. The counts again were highly aggregated hence a negative binomial glm was used for analysis using the glm.nb function in the MASS package of R (Venables & Ripley, 2002).

## 3 Results

### 3.1 Observed mortality rates

High mortality rates were observed in all groups during the trial, nonetheless being markedly lower in SG and HG, than in CG and MG. In general, the lowest mortality rates were observed during week 1 of the trial, except for SG, showing a slightly decreased mortality rate in week 2, compared to week 1. A maximum of mortality was reached at week 3 (Table 2).

**Table 2:** Mortality rates of the different feeding groups.

	Mortality rate (%)			
Week of the trial	CG	SG	HG	MG
Week 1	5.00	6.25	5.00	8.75
Week 2	17.11	4.00	7.89	19.18
Week 3	23.81	13.89	17.14	20.34
Week 4	8.33	9.68	8.62	10.64
Week 1–4	45.00	30.00	33.75	47.50

Table 2 shows the mortality rates of the different feeding groups in the single weeks of the trial (%). CG, Control Group; SG, Sainfoin Group; HG, Herbal Group; MG, Mineral Group.

### 3.2 Mean weights and weight gain

**Fig. 2:**
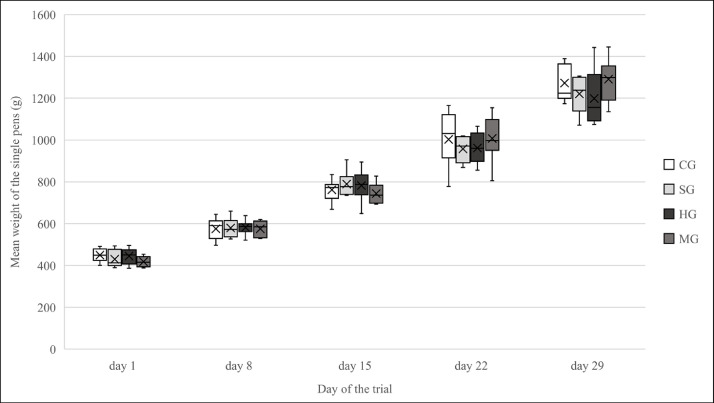
Progression of the weights of the four feeding groups during the trial. Fig. 2 shows the progression of the mean weights per pen, measured on day 1, 8, 15, 22 and 29 of the trial. The box-whisker plots show median, mean and central 50th percentiles. According to the Linear Mixed Model no significant difference in the weight gains of SG (*p* = 0.98), HG (p = 0.58) and MG (p = 0.25) compared to CG; *n* = 80 rabbits/group; CG = Control Group; SG = Sainfoin Group; HG = Herbal Group; MG = Mineral Group.

The observed mean weight gains per pen (SG: p = 0.98; HG: p = 0.58; MG: p = 0.25) of SG, HG and MG were not significantly different from the weight gains of CG. Fig. 2 illustrates the progression of the weights of the fattening rabbits in the different feeding groups over the course of the trial.

### 3.3 Results of laboratory examinations

Digestive diseases were by far the most common cause of death during the entire trial. As anticipated, *Eimeria* sp. and *E. coli* could be confirmed as the most common pathogens in the examined weaning period*.* In all rabbits with *E. coli* dysentery, also *Eimeria* sp. were present in the intestines. The highest incidence of *E. coli* overgrowth (93.88% of all dissected rabbits) was reached at week 3 of the study, one week after the maximum of oocyst shedding. In six cases *E. coli* was the causative agent of septicaemia or was detectable by culture in the lungs, liver or uterus. Indeed, infections with other pathogens (*Cl. perfringens, Pasteurella multocida* and *Staphylococcus aureus*) were detected comparatively rare (Table 3). During the study, *Salmonella* sp. was found neither in the examined faecal samples nor in the intestinal contents. Likewise, no evidence of liver coccidiosis (*E. stiedai*) was detected in smears from gall bladder content.

**Table 3:** Pathogens detected during examination of the perished rabbits.

Detected pathogens during examination of the perished rabbits	Amount of cases found during examination of the perished rabbits (cases/number of perished rabbits)				
	CG	SG	HG	MG	In total
*Eimeria* sp.	36/36 (100%)	24/24 (100%)	27/27 (100%)	36/38 (94.74%)	123/125 (98.40%)
*E. coli*	27/36 (75.00%)	17/24 (70.83%)	23/27 (85.19%)	25/38 (65.79%)	92/125 (73.60%)
*Cl. perfringens*	1/36 (2.78%)	2/24 (8.33%)	0/23 (0.00%)	3/38 (7.89%)	6/125 (4.80%)
*Pasteurella multocida*	3/36 (8.33%)	1/24 (4.17%)	1/27 (3.70%)	1/38 (2.63%)	6/125 (4.80%)
*Staphylococcus aureus*	2/36 (5.56%)	1/24 (4.17%)	0/27 (0.00%)	2/38 (5.26%)	5/125 (4.00%)

Table 3 shows all pathogens, detected during the examination of the perished rabbits. In all groups *Eimeria* sp. was found most frequently, followed by an intestinal overgrowth with *E. coli*. In comparison, infections due to other pathogens occurred rarely. CG, Control Group; SG, Sainfoin Group; HG, Herbal Group; MG, Mineral Group.

### 3.4 Oocysts per gram (OPG) counts in faecal and intestinal samples

**Fig. 3:**
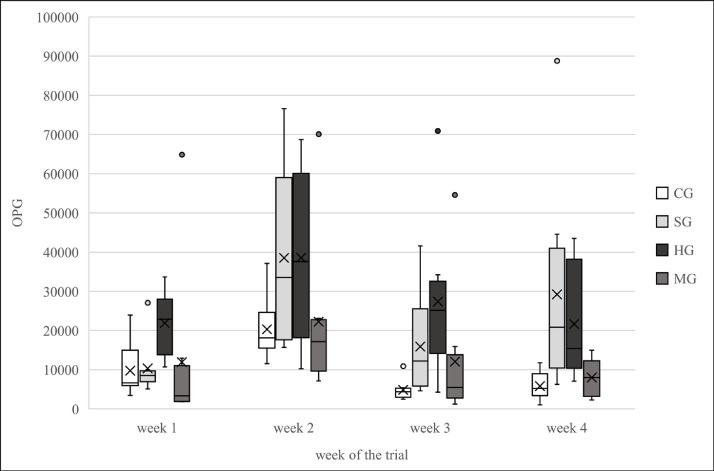
Weekly oocyst output of the four different feeding groups during the trial. Fig. 3 shows the weekly oocyst output per pen of the four different feeding groups of fattening rabbits over the course of the trial, measured as oocysts per gram faeces (OPG). The box-whisker plots show median, mean and central 50th percentiles, the points represent outliers. According to the Negative Binomial Generalized Linear Mixed Model significantly higher oocyst output of  SG (*p* = 1.4E-03) and  HG (*p* = 1.4E-05) than of  CG; No significant difference in oocyst excretion between  CG and  MG (*p* = 0.24); Maximum of OPG counts in faecal samples was reached in week 2; *n* = 80 rabbits/group; CG = Control Group; SG = Sainfoin Group; HG = Herbal Group; MG = Mineral Group.

The statistical evaluation of the faecal samples revealed a significantly higher oocyst output in SG (p = 1.4E-03) and HG (p = 1.4E-05) compared to CG. Whereas in MG, no significant difference in the oocyst output compared to CG could be observed (p = 0.24). The maximum of OPG counts in the faecal samples was reached in week 2 of the trial (Fig. 3).

In the comparison of the slaughtered rabbits at the end of the trial, neither the oocyst content of jejunum and ileum (SG: p = 0.50; HG: p = 0.86; MG: p = 0.75) nor the caecal OPG counts (SG: p = 0.77; HG: p = 0.93; MG: p = 0.13) showed a significant difference between CG and one of the other feeding groups. In pen 5 of CG all rabbits died during the trial. Hence, no rabbit of pen 5 of CG could be sacrificed and examined at the end of the trial (Table 4).

**Table 4:** OPG counts in the intestinal samples of the sacrificed fattening rabbits.

	CG		SG		HG		MG	
	jej & ile	cae	jej & ile	cae	jej & ile	cae	jej & ile	cae
PPen 1	5.70E + 03	1.19E + 05	2.73E + 04	2.67E + 04	1.08E + 04	6.24E + 04	3.63E + 04	4.05E + 04
PPen 2	4.50E + 03	1.68E + 04	9.00E + 03	1.95E + 04	9.00E + 02	2.13E + 04	3.75E + 03	1.38E + 04
PPen 3	5.31E + 04	1.76E + 05	8.40E + 03	4.74E + 04	1.02E + 04	2.22E + 04	5.10E + 03	2.34E + 04
PPen 4	2.40E + 04	5.70E + 04	4.02E + 04	1.12E + 05	9.00E + 03	3.00E + 04	9.30E + 03	1.32E + 04
PPen 5	n.d.^1^	n.d.^1^	4.20E + 03	2.73E + 04	4.35E + 03	3.09E + 04	5.52E + 04	9.36E + 04
PPen 6	2.13E + 04	2.70E + 04	3.23E + 04	4.08E + 04	3.84E + 04	9.36E + 04	4.20E + 03	7.05E + 03
PPen 7	6.00E + 02	4.50E + 02	1.83E + 04	1.04E + 05	3.15E + 04	7.44E + 04	9.00E + 02	1.50E + 03
PPen 8	1.08E + 04	8.70E + 03	5.43E + 04	1.55E + 05	2.01E + 04	1.11E + 05	1.80E + 03	3.30E + 04

Table 4 shows the OPG counts in the intestinal samples of the sacrificed fattening rabbits. According to negative binomial generalized linear mixed model neither the oocyst content of jejunum and ileum (SG: *p* = 0.50; HG: *p* = 0.86; MG: *p* = 0.75) nor the caecal OPG counts (SG: p = 0.77; HG: *p* = 0.93; MG: *p* = 0.13) showed a significant difference between CG and one of the other feeding groups. In pen 5 of CG all rabbits died during the trial. Hence, no rabbit of pen 5 of CG could be examined. ^1^ not done; CG, Control Group; SG, Sainfoin Group; HG, Herbal Group; MG, Mineral Group; jej & ile, jejunum & ileum; cae, caecum; OPG, Oocysts per gram faeces.

## 4 Discussion

### 4.1 Causes of losses in the first four weeks after weaning

During weaning, fattening rabbits are particularly susceptible towards digestive disorders and external stressors. Strong modifications of the diet from maternal milk, which has protective effects against the pathogens, to a diet only consisting of solid feed increases the vulnerability (Barthold et al., 2016; De Blas et al., 2012; Pakandl, 2009).

The results of the study verified the impact of gastrointestinal disturbances in rabbits in the weaning period and in particular the relevance of coccidiosis and *E. coli* overgrow. In comparison, other pathogens only played a minor part. *Eimeria* sp. was the most prominent pathogen, present in all examined rabbits during the study. This is consistent with the opinion of several authors, who point out the widespread occurrence of *Eimeria* sp. in rabbits (Licois, 2004; Okumu et al., 2014; Szkucik et al., 2014). Furthermore, the decline of oocyst excretion after the second week of the trial highlights the critical role of coccidia especially in the first weeks after weaning. Earlier studies also conclude that the parasite becomes less important in older fattening rabbits (Nosal et al., 2014; Okumu et al, 2014; Papeschi, Fichi & Perrucci, 2013). This might be due to a maturation of the GALT (gut associated lymphoid tissue) over time, leading to a higher resistance towards the parasite (Pakandl, 2009).

Although some authors consider a threshold problematic (Pakandl, 2009), others determined a number of 8000 OPG in intestinal contents as high (Okumu et al., 2014). This value was exceeded by 91.13% of the rabbits, that died during the trial and 90.32% of the rabbits, sacrificed at the end of the study. The high prevalence of the parasite measured in the examined rabbitry points to coccidial contamination of the stables in the current field trial, likely because of persistence of oocysts in the environment due to their high tenacity (Licois, 2004).

As described by Barthold et al. (2016), *Eimeria* sp., apart from being pathogenic itself, has a pioneering role of causing alterations in the intestinal pH, which allows *E. coli* to overgrow. Indeed, an infection with *Eimeria* sp. was mostly combined with an overgrowth of *E. coli* in the current study. Another interesting finding is that in- respectively decreases in oocyst shedding were always followed by in- respectively decreases in the case numbers of *E. coli* dysentery and mortality rates. These findings affirm the importance of controlling coccidiosis, being the first step in the onset of intestinal disease, as a prerequisite to also control *E. coli* dysentery and mortality. This accords with earlier observations, which showed that the clinical severity of coccidiosis is substantially enhanced by secondary bacterial infections (Kowalska, 2012).

### 4.2 Improvement of the health status of weaned rabbits by feed supplements

Coccidiostats like robenidine are often used to control coccidiosis and mortality after weaning in rabbits. Although the positive impact of robenidine on coccidiosis is proven (Coudert, 2004; Vancraeynest, De Gussem, Marien & Maertens, 2008), there is also concern about development of resistance mechanisms or an insufficient effectiveness of the coccidiostat against *Eimeria* sp. (Coudert, 2004; Kowalska et al., 2012). The high mortality rates and OPG counts in the feeding groups receiving robenidine, showed that the effectiveness was not satisfactory in the examined rabbitry either. The markedly lower mortality rates in SG and HG show, that the plant additives might be a useful addition or even alternative to robenidine in controlling mortality in weaned fattening rabbits, especially because the development of antimicrobial resistances to plants is very unlikely. In contrast to synthetical drugs whose effectivity usually results from one distinctive active substance, plants act as multi target drugs based on the complex synergism of the compounds (Huang, Liu, Zhao, Hu & Wang, 2017). One limitation of the study is, that sainfoin was only supplemented in combination with robenidine. Further studies are needed to determine its effectiveness as a single supplement. An addition of the herbal mixture with clinoptilolite seems to impair the effects of the herbs, which can be seen in higher mortality rates in MG compared to HG.

At first sight, the significantly higher oocyst excretion in SG and HG during the trial seems to be inconsistent to the measured mortality rates and the hypothesis of an improvement of the intestinal health by sainfoin or the herbal mixture. But interestingly, the four different diets did not cause a significant difference in the OPG counts amongst the intestinal samples of the four groups of sacrificed rabbits at the end of the trial. This phenomenon might be explained by higher surviving rates of diseased rabbits in SG and HG, which thus led to a higher overall excretion of oocysts in the two groups during the trial.

However, the lower mortality in SG and HG might also be explained by inhibitory effects of plant compounds on *E. coli* at least partly preventing intestinal overgrowth and thus higher mortality rates. Sainfoin, and the tannins contained within (Dalle Zotte & Cossu, 2009; Liu et al., 2013), as well as garlic (Ankri & Mirelman, 1999) and conessi tree (Siddiqui et al., 2012) have documented effects against *E. coli*. Concerning overall rabbit health, positive effects of tannin enriched diets (Dalle Zotte & Cossu, 2009; Legendre et al., 2018) and the supplementation of garlic (in combination with oregano) (Kowalska et al., 2012) were already shown.

A note of caution in the interpretation of the results is due to the considerable influence of environmental stressors and high infection pressure, showing in high OPG counts and mortality rates amongst all feeding groups. High mortality rates in CG and MG, might also partly be explained by their location alongside the walls of the building. Cold stress leads to disruptions of the protective intestinal microflora, reducing the resilience of the rabbits and very probably fostering high mortality rates (Fortun-Lamonthe & Boullier, 2004; Wang et al., 2019). Previous studies also stressed the importance of hygiene and proper housing conditions, such as avoiding cold stress, as well as the control of chronic diseases, to prevent intestinal diseases in rabbits (Kowalska et al, 2012; Wang et al., 2019). Thus, the feed supplements should be investigated further under optimised conditions, to determine their usefulness in the field.

## 5 Conclusion

In conclusion, the predominant role of *Eimera* sp. and *E. coli* in rabbit weaners and the need for alternative strategies next to robenidine, to manage mortality could be confirmed in the field. Although promising effects of sainfoin silage and Herb-All COCC-X could be observed, high mortality rates and oocyst excretion in all four feeding groups were detected. Thus, lower infection pressure and a lower influence of external stressors is mandatory, before rabbitries can sufficiently benefit from the feed supplements in the field. Further studies under optimised conditions are required, to detect possible further effects of the natural supplements, which might have been masked by the prevailing circumstances in the current study.

## Ethical statement

The authors confirm that the ethical policies of the journal, as noted on the journal's author guidelines page, have been adhered to. Ethical approval was required and the study was conducted formally approved by the Veterinary office of the Canton of Aargau (License no. 75724).

## Declaration of Competing Interest

The authors declare that they have no known competing financial interests or personal relationships that could have appeared to influence the work reported in this paper.
